# Does Forward Head Posture Influence Muscle Tone, Stiffness, and Elasticity in University Students?

**DOI:** 10.3390/jcm14061888

**Published:** 2025-03-11

**Authors:** Min-Sik Yong, Hae-Yong Lee

**Affiliations:** Department of Physical Therapy, Youngsan University, 288, Junam-ro, Yangsan-si 50510, Gyeongnam, Republic of Korea; yongms@ysu.ac.kr

**Keywords:** forward head posture, muscle tone, muscle stiffness, muscle elasticity

## Abstract

**Background/Objectives**: The present study aimed to investigate the relationship between forward head posture (FHP) and the mechanical properties of muscles as well as the influence of FHP on them. **Methods**: To define participants with FHP, craniovertebral angle (CVA) was measured. All participants were divided into two groups in accordance with their CVA: the experimental group (FHP) consisting of participants with a CVA below 50°, and the control group (CON) consisting of participants with a CVA above 50°. The tone, stiffness, and elasticity of the upper trapezius muscle (UT), the middle trapezius muscle (MT), the lower trapezius muscle (LT), the sternocleidomastoid muscle (SCM), the splenius capitis muscle (SC), the pectoralis major muscle (PM), and the serratus anterior muscle (SA) were measured using MyotonPro (Myoton AS, Tallinn, Estonia). **Results**: Both tone and stiffness in the UT were statistically significant (*p* < 0.05). In addition, stiffness in the LT was statistically significant (*p* < 0.05). No significant differences were found in tone, stiffness, and elasticity of the MT, SCM, SC, PM, and SA muscles (*p* > 0.05). A significant correlation was found between FHP and both tone and stiffness in the UT (r = −0.731, *p* = 0.000; r = −0.749, *p* = 0.000, respectively). No significant correlation was found between FHP and tone, stiffness, and elasticity of the MT, LT, SCM, SC, PM, and SA muscles. **Conclusions**: Since the UT was the muscle in which changes in mechanical properties were first induced by FHP, an approach targeting UT is necessary as a priority when treating patients with FHP.

## 1. Introduction

Smartphones have become an essential tool in the modern world; people spend a lot of time using them in their daily lives. As the spread and use of these smart products increases, the incidence of various musculoskeletal disorders also rises [1]. Poor posture, for example, forward head posture (FHP), is a common concern in office workers, and the popularization of smartphones has led to abnormal neck and shoulder alignment caused by FHP in many people [2,3,4].

In FHP, there is a relationship between excessive extension of the upper cervical spine and shortening of the upper trapezius (UT), cervical extensor muscles, sternocleidomastoid (SCM), and the levator scapulae muscle [3,5]. FHP can lead to development of several musculoskeletal problems, and people with FHP have higher activation of neck muscles compared to those without FHP [6,7,8]. However, not all people with FHP have clinical symptoms [9,10].

Many studies have been conducted on muscle activity related to FHP. Mechanical properties are also important indicators of the health of the musculoskeletal system. Elasticity is the body’s ability to recover its previous configuration after being deformed by a load. The higher the elasticity, the greater the body’s ability to return to its original shape [11]. Myofascial stiffness is one of the essential indicators of energy storage in the muscle–tendon unit and can affect the control of joint motion. As BMI increases, anterior head posture increases, and this has been associated with muscle stiffness and elasticity [12]. In addition, stiffness related to muscle fatigue increases after periods of work [13] and ergonomic risks are strongly associated with increased stiffness and increased muscle tone [14].

Patients with shoulder and neck pain have high stiffness in the trapezius muscle, and accumulated fatigue in the trapezius muscle can cause injury to the neck muscles [15,16]. This is also related to the range of rotational movement of the cervical spine and stiffness of the upper trapezius muscle [17]. The mechanical parameters of SCM have a greater influence on passive structural properties than the UT and are better predictors of biological aging [12]. In addition, most of the variability in the stiffness and elasticity parameters is explained by age. This way, mechanical parameters such as stiffness and elasticity are considered predictive factors for diseases and disorders.

Although there are many studies on the effects of FHP on muscles, there is only one study showing that FHP has no impact on muscle stiffness, tone, and elasticity [18], and there is insufficient explanation of the mechanical properties of muscles known to be affected by FHP. As FHP affects muscle activity around the neck region, it will affect mechanical properties such as muscle elasticity, stiffness, and tension. As a result, it was hypothesized that FHP could lead to changes in mechanical properties that can lead to dysfunction of the musculoskeletal system. In future, assessments of stiffness and elasticity of the muscle may provide important information for the evaluation and planning of specific therapeutic interventions to improve muscle function in the neck region.

This study aims to investigate the influence of FHP on the mechanical properties of muscles in university students in their 20s and to increase understanding of the musculoskeletal imbalances and disorders caused by FHP.

## 2. Materials and Methods

### 2.1. Participants

The present study involved 24 healthy university students who met the inclusion criteria (Table 1). The study inclusion criteria were as follows: (1) no history of neurological surgery or injuries, (2) no history of musculoskeletal surgery or injuries, (3) no pathological musculoskeletal dysfunctions, i.e., spinal deformities such as scoliosis and kyphotic deformities of the spine as a consequence of serious disease such as ankylosing spondylitis, and (4) no acute and chronic pain syndromes in the region of the neck. A total of 32 participants meeting the inclusion criteria responded to the study announcement, and 8 men who did not meet the inclusion criteria were excluded after an interview with each participant. Since all participants who applied for this study were men, only healthy men over 20 years of age and without pain in the cervical spine and neck in the past 3 months were recruited into this study. To eliminate potential sources of bias, the measurements of mechanical properties of muscles were performed in a separate air-conditioned light room between 9 a.m. and noon. Each participant signed an informed consent form prior to study initiation. The study was conducted between April 2021 and November 2021. The local and central ethical committees of Youngsan University approved the study (YSUIRB-202101-HR-084-02). The study adhered to the principles of the Declaration of Helsinki.

**Figure 1:**
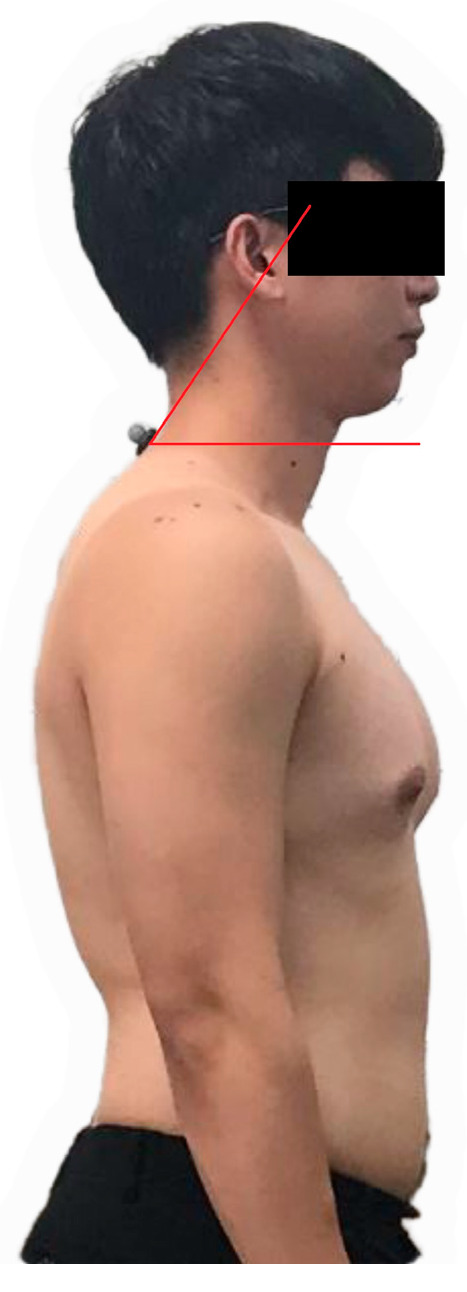
Craniovertebral angle.

The craniovertebral angle (CVA) was measured using a photometric method, with the participants in the standing position (Figure 1). Before taking the image, the C7 process and the tragus were marked. A camera was placed 1.5 m away from the lateral surface of the body at the height of the acromial process. Each participant was requested to stand in their natural position and look at a point highlighted on the wall for a few seconds. A photo of the upper part of the body was taken from the side when participants were standing in the natural position. Before taking the measurement, participants were asked for cervical flexion and extension to adjust to a natural head position [18]. The angle between the horizontal line passing through C7 and the line extending from the tragus to C7 is the CVA [19]. A CVA of below 50° is regarded as indicative of forward head posture (FHP) [20]. The participants were divided into two groups according to their CVA: the experimental group (FHP) consisting of participants with a CVA below 50°, and the control group (CON) consisting of participants with a CVA above 50°.

### 2.2. Protocol

Measurements of the mechanical properties, including tone, stiffness, and elasticity of muscles, were taken to assess objective data. The upper trapezius muscle (UT), the middle trapezius muscle (MT), the lower trapezius muscle (LT), the sternocleidomastoid muscle (SCM), the splenius capitis muscle (SC), the pectoralis major muscle (PM), and the serratus anterior muscle (SA) were examined using MyotonPro (Myoton AS, Tallinn, Estonia) in a static upright standing position. Myotonometric assessment, a method based on using MyotonPro, provides objective data with regard to mechanical properties of muscles, including tone, stiffness, and elasticity [21]. A previous study using MyotonPro reported that its intra-rater reliability was excellent (intra-class correlation coefficients, ICC > 0.99) [22]. The probe of the device (3 mm in diameter) was placed perpendicular to the surface of the skin in the muscular belly and the values of the tone (Hz), stiffness (N/m), and elasticity (%) of muscles were examined by analyzing muscle oscillations in response to brief impulses (0.4 N for 15 ms) with a constant preload (0.18 N). The measurements were taken 3 times for each muscle by the same physical therapist and the average of these measurements was recorded [12,21].

### 2.3. Data Analysis

A quick Fourier transform according to established formulas was used to calculate the maximum oscillation frequency of soft tissues (Hz), indicating muscle tone from the accelerometer data spectrum. The highest acceleration of soft tissue oscillation and mass of probe divided by the maximum displacement of the soft tissue oscillation was used to calculate muscle stiffness. The logarithmic decrement in natural tissue oscillation generated with a single mechanical impulse was used to calculate elasticity [23].

### 2.4. Statistical Analysis

The Shapiro–Wilk test was used for normality evaluation to analyze the distribution of the data. According to the results of the test, the significance of the differences in the average values of FHP and CON was determined using the parametric independent samples *t* test if the data were normally distributed and variances were equal. However, the nonparametric test (Mann–Whitney U test) was performed if the distribution was different than normal and the variance was nonuniform. Pearson’s correlation coefficients were used to determine the relationship between FHP and tone, stiffness, and elasticity of muscles. Statistical analyses were performed using SPSS version 25.0 (IBM Corp., Armonk, NY, USA), and statistical significance was set at *p* < 0.05.

## 3. Results

**Figure 2:**
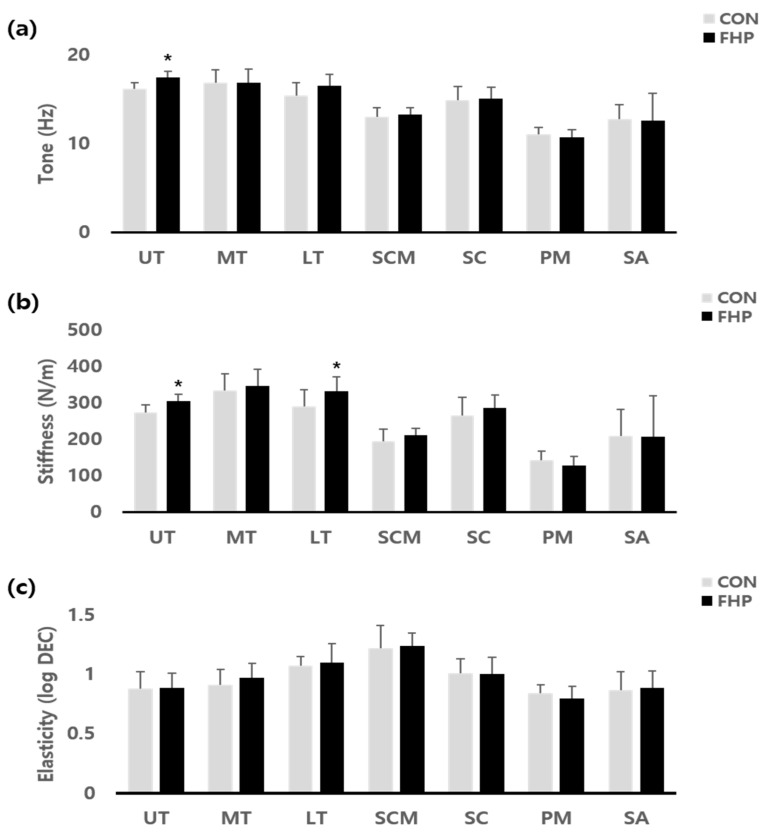
Mechanical properties of muscles: (**a**) tone; (**b**) stiffness; (**c**) elasticity; * *p* < 0.05.

Demographic data of participants are summarized in Table 1. Regarding the effects of FHP on tone, stiffness, and elasticity of muscles, including the upper trapezius muscle (UT), the middle trapezius muscle (MT), the lower trapezius muscle (LT), the sternocleidomastoid muscle (SCM), the splenius capitis muscle (SC), the pectoralis major muscle (PM), and the serratus anterior muscle (SA), both tone and stiffness in the UT were statistically significant (*p* < 0.05). In addition, stiffness in the LT was statistically significant (*p* < 0.05). No significant differences were found in tone, stiffness, and elasticity of the MT, SCM, SC, PM, and SA muscles (*p* > 0.05) (Table 2) (Figure 2).

With respect to the correlations between forward head posture (FHP) and tone, stiffness, and elasticity of the UT, MT, LT, SCM, SC, PM, and SA, a significant correlation was found between FHP and both tone and stiffness in the UT (r = −0.731, *p* = 0.000; r = −0.749, *p* = 0.000, respectively). No significant correlation was found between FHP and tone, stiffness, and elasticity of the MT, LT, SCM, SC, PM, and SA muscles (Table 3).

## 4. Discussion

The aim of the present study was to investigate the relationship between forward head posture (FHP) and the mechanical properties of the upper trapezius muscle (UT), the middle trapezius muscle (MT), the lower trapezius muscle (LT), the sternocleidomastoid muscle (SCM), the splenius capitis muscle (SC), the pectoralis major muscle (PM), and the serratus anterior muscle (SA), as well as the influence of FHP on them. The results demonstrated that FHP could affect both tone and stiffness in the UT. In addition, FHP affected LT stiffness. However, no significant differences were found when comparing tone, stiffness, and elasticity in the MT, SCM, SC, PM, and SA muscles between CON and FHP. There was a significant negative correlation between FHP and UT tone and stiffness. However, there was no significant correlation between FHP and mechanical properties in MT, LT, SCM, SC, PM, and SA muscles.

FHP is a typical neck-related disorder that is observed in people who spend significant amounts of time performing sedentary work [24]. If the condition where the position of the head is shifted forward in relation to the trunk is maintained, the result could be shortening of the cervical extensors and tightening of the cervical flexors. Furthermore, the muscle imbalance observed in FHP caused changes not only in the strength of muscles but also in their electromyographic activities [18,25,26]. The biomechanical parameters in the mechanical properties of the muscles have been investigated in many studies in recent years [11,27,28,29]. A previous study measuring the tone, stiffness, and elasticity of the UT in women in their 20s showed that tone and stiffness significantly increased and elasticity significantly decreased in the sitting position compared to the lying position [30]. Furthermore, in a previous study that measured the mechanical properties of UT using computer operators in their 20s, significant differences in tone and stiffness were observed in the sitting position but elasticity was not significantly different [15]. These results demonstrate that sitting posture affects changes in the mechanical properties of UT. The results of the present study, which measured tone, stiffness, and elasticity in the muscles of university students in their 20s who spend a lot of time sitting, show significant differences in the tone and stiffness of UT; therefore, they are partially consistent with the results of the other studies mentioned above. Additionally, it is assumed that FHP, which could be caused by a prolonged sitting posture, affected mechanical properties.

The increased moment arm of the cervical extensors to counteract the moment of inertia of the head, including the UT, results from FHP [5]. In addition, it has been reported that people with FHP exhibit increased UT electromyographic activity [3]. According to Kocur et al.(2017) [30], significant increases in the tone, stiffness, and elasticity of the UT were caused by increased muscle activation during the transition from lying to sitting positions. From the aforementioned facts, it is suggested that the increased tone and stiffness of the UT results from greater UT activity in the FHP group compared to the CON group. In addition, the results of the present study, which show a significant increase in the tone and stiffness of the UT, suggest that FHP may lead to a variety of musculoskeletal problems by negatively affecting the normal function of muscles. This is consistent with previous studies, which have reported that the mechanical properties of individual muscles reflect their actual static function [30].

In the present study, no significant differences were found in the tone and stiffness of the MT, SCM, SC, PM, and SA muscles between the FHP and CON groups. In a previous study, it was reported that FHP induced changes in the muscle tendon unit length of those muscles [31]. However, the results of the present study showed that differences in tone and stiffness in those muscles were not statistically significant. These results could be explained by the fact that the subjects recruited for the present study did not have severe FHP. Since there was not much difference in average CVA between the FHP and CON groups, it is suggested that this might not be enough to induce significant differences in mechanical properties of the MT, SCM, SC, PM, and SA muscles. Although the relationships between FHP and variables such as neck disorder, muscle activity, chronic neck pain, tension-type headache, etc., have been extensively studied, they have not been clearly defined [10,32,33,34]. It is thought that FHP might not necessarily affect the mechanical properties of muscles and that the changes in tone, stiffness, and elasticity might depend more on the variables [18]. Thus, additional research is necessary to confirm the relationship between muscle mechanical properties and other variables.

All participants recruited in the present study were university students in their 20s. It is quite natural for university students to spend most of their time using either a cell phone or a computer in a sitting position. Since the UT plays an important role in maintaining the position of the head during sitting, the activity of the upper trapezius muscle could easily increase according to the forward movement of the head [35]. Pain in the cervical and shoulder region could be produced if the UT is activated for a long period of time. This suggests that there might be a possible association between the UT overload and chronic pain [23]. Furthermore, it is demonstrated that strength, coordination, and stabilizing function of the UT could be reduced by sitting [36,37]. Therefore, it is suggested that the UT should be the first consideration when applying interventions to FHP patients without other musculoskeletal-related clinical symptoms, because these factors could influence the role of the UT in maintaining natural head posture. Thus, it is recommended that university students who spend a lot of time sitting take short breaks to reduce changes in the mechanical properties of the UT.

The present study showed that FHP was negatively correlated with tone and stiffness in the UT. However, no significant correlation was found between FHP and the mechanical properties of the MT, LT, SCM, SC, PM, and SA muscles. These results suggest that the upper trapezius muscle may be the most vulnerable to changes in CVA. Additionally, as mentioned above, it is possible that tone, stiffness, and elasticity were influenced by variables other than FHP [18]. UT tone and stiffness were significantly correlated with FHP; however, for muscles with no FHP correlation, the influence of other variables, including muscle fatigue, physical activity, pain history, etc., on muscle parameters needs to be considered. Although individual differences or long-term effects were not considered in this study, it is noteworthy that the UT was the muscle in which changes in mechanical properties were first induced by FHP in male students in their 20s.

The limitations of the present study are as follows: First, the results of the present study are not generalizable, because all participants were men. Second, we did not measure the activities of all upper limb muscles affected by FHP. Third, the participants in the FHP group did not have severe FHP. It is likely that severe FHP would affect the mechanical properties of neck muscles. Consequently, the limitations mentioned above should be considered in future studies to clarify the relationship between FHP and the mechanical properties of the muscles.

## 5. Conclusions

This study showed that FHP increases the tone and stiffness of the UT and the stiffness of the LT in male university students in their 20s. Furthermore, FHP was negatively correlated with both tone and stiffness in the UT. However, there were no significant differences in tone, stiffness, and elasticity in the MT, SCM, SC, PM, and SA muscles between groups. In addition, there was no relationship between the tone, stiffness, and elasticity in the MT, LT, SCM, SC, PM, and SA muscles. These findings demonstrate that the UT is the muscle in which changes in mechanical properties are first induced by FHP. Therefore, our data suggest that an approach targeting UT is necessary as a priority when treating patients with FHP.

## Figures and Tables

**Table 1:** General characteristics of the participants.

Variables	FHP (*n* = 13)	CON (*n* = 11)
Age (years)	21.87 ± 0.83	21.57 ± 1.39
Height (cm)	172.25 ± 4.89	176.28 ± 5.18
Weight (kg)	69.62 ± 6.09	68.74 ± 5.42
CVA (°)	47.46 ± 1.31	57.08 ± 3.90

All values are mean ± standard deviation.

**Table 2:** Effects of FHP on tone, stiffness, and elasticity of muscles.

Muscle	Variables	FHP	CON	t	*p*
UT	tone	17.41 ± 0.70	16.11 ± 0.70	4.49	0.00 *
	stiffness	304.16 ± 21.55	272.48 ± 19.82	3.72	0.00 *
	elasticity	0.89 ± 0.14	0.88 ± 0.12	0.03	0.97
MT	tone	16.83 ± 1.46	16.85 ± 1.57	−0.03	0.97
	stiffness	346.06 ± 45.50	332.96 ± 45.20	0.70	0.48
	elasticity	0.97 ± 0.13	0.91 ± 0.12	1.62	0.11
LT	tone	16.51 ± 1.44	15.39 ± 1.29	1.98	0.06
	stiffness	331.99 ± 45.66	290.75 ± 39.37	2.34	0.02 *
	elasticity	1.10 ± 0.08	1.07 ± 0.16	0.55	0.58
SCM	tone	13.25 ± 1.01	13.02 ± 0.75	0.62	0.54
	stiffness	210.85 ± 31.99	195.24 ± 19.30	1.41	0.17
	elasticity	1.24 ± 0.19	1.22 ± 0.11	0.34	0.73
SC	tone	15.07 ± 1.51	14.91 ± 1.21	0.29	0.77
	stiffness	284.70 ± 51.63	264.03 ± 36.30	1.11	0.27
	elasticity	1.00 ± 0.12	1.01 ± 0.14	−0.28	0.78
PM	tone	10.67 ± 0.72	11.05 ± 0.88	−1.15	0.26
	stiffness	127.06 ± 23.09	143.36 ± 24.73	−1.66	0.10
	elasticity	0.80 ± 0.07	0.84 ± 0.10	−1.22	0.23
SA	tone	12.60 ± 1.63	12.76 ± 3.06	−0.15	0.87
	stiffness	206.60 ± 74.38	207.93 ± 111.37	−0.03	0.97
	elasticity	0.89 ± 0.15	0.87 ± 0.14	0.26	0.79

All values are mean ± standard deviation. Abbreviation: UT, upper trapezius; MT, middle trapezius; LT, lower trapezius; SCM, sternocleidomastoid; SC, splenius capitis; PM, pectoralis major; SA, serratus anterior. * *p* < 0.05.

**Table 3:** Pearson correlation between FHP and tone, stiffness, and elasticity of muscles.

Muscle	Variables	*R*	*p*
UT	tone	−0.731	0.000 **
	stiffness	−0.749	0.000 **
	elasticity	−0.143	0.506
MT	tone	0.031	0.886
	stiffness	−0.154	0.472
	elasticity	−0.369	0.076
LT	tone	−0.303	0.150
	stiffness	−0.369	0.076
	elasticity	−0.245	0.249
SCM	tone	−0.123	0.566
	stiffness	−0.286	0.175
	elasticity	−0.148	0.491
SC	tone	−0.121	0.573
	stiffness	−0.301	0.153
	elasticity	−0.022	0.920
PM	tone	0.329	0.116
	stiffness	0.311	0.140
	elasticity	0.077	0.721
SA	tone	0.105	0.624
	stiffness	0.057	0.793
	elasticity	−0.074	0.732

All values are mean ± standard deviation. Abbreviation: UT, upper trapezius; MT, middle trapezius; LT, lower trapezius; SCM, sternocleidomastoid; SC, splenius capitis; PM, pectoralis major; SA, serratus anterior. ** *p* < 0.01.

## Data Availability

The data presented in this study are available in the article.

## Abbreviations

The following abbreviations are used in this manuscript:

FHP	Forward Head Posture
UT	Upper Trapezius
SCM	Sternocleidomastoid
CVA	Craniovertebral angle
MT	Middle Trapezius
LT	Lower Trapezius
SC	Splenius Capitis
PM	Pectoralis major
SA	Serratus Anterior
